# Delta inflation: a bias in the design of randomized controlled trials in critical care medicine

**DOI:** 10.1186/cc8990

**Published:** 2010-04-29

**Authors:** Scott K Aberegg, D Roxanne Richards, James M O'Brien

**Affiliations:** 1 Department of Critical Care, Jordan Valley Medical Center, 3580 West 9000 South, West Jordan, Utah, 84088, USA; 2 Department of Family Medicine, University of Virginia Health System, 1215 Lee Street, Charlottesville, Virginia, 22908, USA; 3 Department of Internal Medicine, The Ohio State University College of Medicine, 410 West 10th Avenue, Columbus, Ohio, 43210, USA

## Abstract

**Introduction:**

Mortality is the most widely accepted outcome measure in randomized controlled trials of therapies for critically ill adults, but most of these trials fail to show a statistically significant mortality benefit. The reasons for this are unknown.

**Methods:**

We searched five high impact journals (Annals of Internal Medicine, British Medical Journal, JAMA, The Lancet, New England Journal of Medicine) for randomized controlled trials comparing mortality of therapies for critically ill adults over a ten year period. We abstracted data on the statistical design and results of these trials to compare the predicted delta (delta; the effect size of the therapy compared to control expressed as an absolute mortality reduction) to the observed delta to determine if there is a systematic overestimation of predicted delta that might explain the high prevalence of negative results in these trials.

**Results:**

We found 38 trials meeting our inclusion criteria. Only 5/38 (13.2%) of the trials provided justification for the predicted delta. The mean predicted delta among the 38 trials was 10.1% and the mean observed delta was 1.4% (*P *< 0.0001), resulting in a delta-gap of 8.7%. In only 2/38 (5.3%) of the trials did the observed delta exceed the predicted delta and only 7/38 (18.4%) of the trials demonstrated statistically significant results in the hypothesized direction; these trials had smaller delta-gaps than the remainder of the trials (delta-gap 0.9% versus 10.5%; *P *< 0.0001). For trials showing non-significant trends toward benefit greater than 3%, large increases in sample size (380% - 1100%) would be required if repeat trials use the observed delta from the index trial as the predicted delta for a follow-up study.

**Conclusions:**

Investigators of therapies for critical illness systematically overestimate treatment effect size (delta) during the design of randomized controlled trials. This bias, which we refer to as "delta inflation", is a potential reason that these trials have a high rate of negative results.

"*Absence of evidence is not evidence of absence."*

## Introduction

Mortality has become the standard outcome measure in trials of therapies in critically ill adults because it obviates debate about clinical relevance and concerns of ascertainment bias. However, it has recently been noted that the majority of these trials fail to demonstrate efficacy [1] and several therapies that appeared promising did not demonstrate efficacy on repeated study [2-7]. The high rate of negative results in these trials could be explained by several possibilities including true lack of efficacy (the null hypothesis is true), type II statistical errors in trials with adequate power, and methodological problems in study design leading to inadequate power and sample size [8].

Several parameters must be chosen by investigators in the design of a trial of mortality in order to determine the required sample size, including the significance level required for rejection of the null hypothesis; power; the predicted mortality rate in the placebo arm; and the predicted effect size (delta). In contrast to significance level and power, which are usually set by convention at 0.05 and 90%, respectively, predictions about the placebo mortality rate must be guided by preliminary data (if available) or guesswork. Likewise, predictions of delta are either based on existing data or are guided by biological plausibility or a minimal clinically important difference (MCID) [9,10]. Using these four variables (significance level, power, baseline mortality rate, and delta) sample size required for the trial can be calculated.

Unfortunately, sample size is often not determined in this fashion [11-13]. As a result of financial, time, and logistical constraints [14], investigators often first estimate the number of patients that they can expect to enroll during the planned duration of the trial with available resources. Then, using conventional values for significance level and power, they calculate the delta that they can expect to find using that sample size, in effect performing sample size calculations in reverse. (It is also not unusual for investigators to revise delta upward mid-trial when declining enrollment is noted [15,16].) As a result of this, values of predicted delta used by investigators in study design may not represent a realistic estimate of the effect of a therapy on outcomes. As shown in Table 1, sample size determinations are much more sensitive to changes in delta than the other three variables; this fact, combined with inflexibility with regard to significance level and power (due to convention), may make delta more susceptible to misuse and manipulation. We refer to biased overestimates of effect size during trial design as 'delta inflation'. If it exists, delta inflation may result in trials that have inadequate sample size to find true differences between a therapy and placebo, leading to a high rate of falsely negative trials, with many attendant implications for critical care research and practice.

**Table 1:** Simulated scenarios for sample size determination in the design of a hypothetical study

	Standard Scenario	Relaxed significance level	Relaxed Power	Baseline Mortality shifted away from 50%	Inflated delta
Significance level (two-sided)	0.05	**0.1**	0.05	0.05	0.05
Power	90%	90%	**80%**	90%	90%
Baseline (placebo) mortality rate	50%	50%	50%	**40%**	50%
Delta (ARR)	10%	10%	10%	10%	**15%**
Required sample size	**1076**	**884**	**816**	**992**	**480**

Inflation of delta has a substantially larger impact on required sample size than changes in the other variables. ARR, absolute risk reduction.

## Materials and methods

One author (SKA) performed a search of the tables of contents of five high-impact medical journals (*BMJ*, *New England Journal of Medicine*, *Journal of the American Medical Association*, *Lancet*, *Annals of Internal Medicine*) for titles containing the keywords (and variations thereof) critically ill, intensive care, ICU, acute respiratory distress syndrome, acute lung injury, sepsis, shock, ventilator, ventilation, respiratory failure, multiple organ dysfunction, continuous veno-venous hemodialysis, and renal failure, but not containing keywords related to pediatrics (neonatal, infant, children, prematurity) published between 1 January, 1999 and 22 July, 2009. Articles containing included keywords were then reviewed further to determine if they met inclusion and exclusion criteria. Articles were included if they described a randomized controlled trial in a critically ill adult population that evaluated proportional mortality (mortality expressed as a proportion as opposed to that measured as a mean survival or a time to event analysis) as the primary endpoint upon which power calculations were based. Articles were excluded if they described a non-inferiority trial, if they dealt with a non-ICU population (out of hospital, pre-hospital, or care not described as delivered in an ICU setting), and if they included non-adult patients. Factorial trials testing more than one therapy were considered as separate trials for each therapy tested, even if reported in the same manuscript.

Data were abstracted from articles meeting these criteria utilizing a standardized form. We recorded variables pertaining to statistical methods including significance level, power, delta, the expected baseline (placebo or standard care) mortality rate, the *a priori *sample size, whether the study was terminated early, and any modifications made to the sample size in the middle of the trial. We recorded whether the predicted delta was justified by reference to either published or unpublished data. We abstracted data from the results of the trial including the number of patients in the treatment and placebo arms that were included in the final data analysis, and the mortality rate in each arm. We recorded unadjusted results and those pertaining to the overall (intention-to-treat) population (so that the results would correspond to the assumptions of the power calculations) even where the authors emphasized adjusted or subgroup analyses. For three trials that did not report the predicted delta, we contacted the authors to obtain this information. For one of these trials [17], the predicted delta could not be determined and the study was excluded. For the other two trials, the authors provided information about the predicted delta and sample size calculations not included in the original manuscript.

Using these data, we performed confirmatory sample size calculations for each trial, determined the observed treatment effect (delta) and the difference between the predicted and observed delta (the delta-gap), calculated the 95% confidence interval for the observed delta, and plotted a graph of observed versus predicted delta. We calculated mean predicted and observed delta values across all trials, and compared them using a paired t-test with unequal variances. For non-statistically significant trials that had an observed delta greater than the smallest predicted delta of all the trials (3% [18]), we calculated the sample size that would be required if the trials were to be repeated using the observed delta of the index trial as the predicted delta for the future trial. All statistical calculations were performed using STATA version 8.0 (College Station, TX, USA).

## Results

Our search identified 160 articles for further review. Of these, 58 described trials that were not randomized controlled trials, 46 were excluded because mortality was not the primary outcome on which power calculations were based, 12 were excluded because they dealt with non-critically ill populations, 2 were excluded because they described non-inferiority trials, 1 was excluded because it dealt with pediatric patients, and 1 was excluded because no predicted delta was reported and the authors could not provide the information. The remaining 38 articles were included in our analysis.

Additional file 1 shows the characteristics of the included trials. Among all trials, only 5 of the 38 (13.2%) provided justification for the predicted delta, and 7 of the 38 (18.4%) provided justification for the baseline mortality rate used in sample size calculations (data not shown). Among all included trials, 27 of the 38 (71%) provided sufficient information for us to replicate the sample size calculations. For 20 of these 27 trials (74%), our sample size calculations yielded values that deviated less than 10% from the *a priori *sample sizes specified in the manuscript.

**Figure 1:**
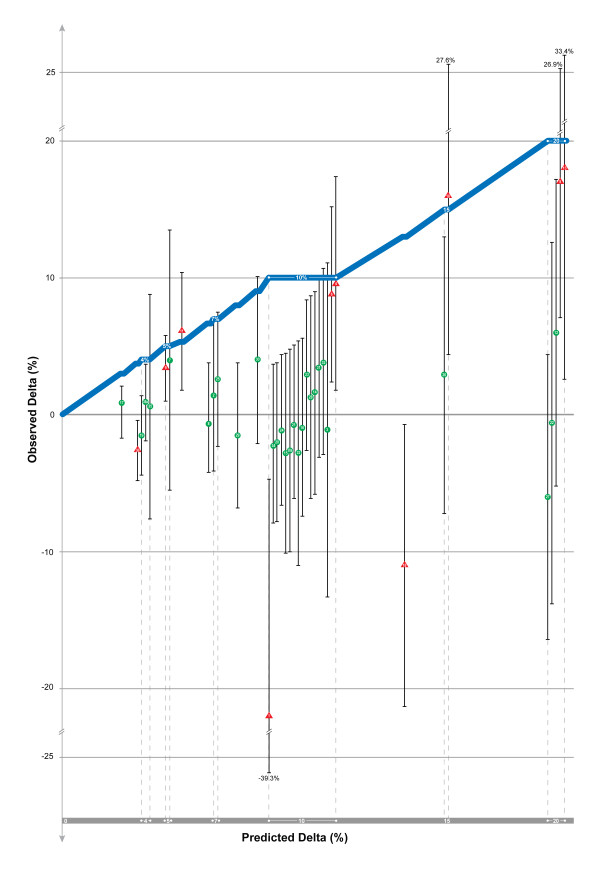
**Plot of observed versus predicted delta (with associated 95% confidence intervals for observed delta) of 38 trials included in the analysis**. Point estimates of treatment effect (deltas) are represented by green circles for non-statistically significant trials and red triangles for statistically significant trials. Numbers within the circles and triangles refer to the trials as referenced in Additional file 1. The blue 'unity line' with a slope equal to one indicates perfect concordance between observed and predicted delta; for visual clarity and to reduce distortions, the slope is reduced to zero (and the x-axis is horizontally expanded) where multiple predicted deltas have the same value and where 95% confidence intervals cross the unity line. If predictions of delta were accurate and without bias, values of observed delta would be symmetrically scattered above and below the unity line. If there is directional bias, values will fall predominately on one side of the line as they do in the figure.

Figure 1 demonstrates graphically the main results of our analysis comparing predicted and observed delta. As seen in Figure 1, values for observed delta are not randomly scattered around the blue line representing unity with predicted delta, but rather fall almost uniformly below it. Among all included trials, only 2 (5.3%) demonstrated an observed delta equal to or greater than the predicted value [19,20]. The mean predicted delta among all trials was 10.1%, the mean observed delta was 1.4% (*P *< 0.0001 for this comparison), and the mean difference between predicted and observed delta (the delta-gap) was 8.7%. Among all trials, only 7 of the 38 included studies (18.4%) demonstrated an unadjusted delta for the intention-to-treat population that was statistically significant in the hypothesized direction (red triangles above zero on the Y-axis in Figure 1). Among all trials, 26 of 38 (68.4%) had 95% confidence intervals for observed delta that did not include the predicted delta, in essence excluding an effect of the therapy as great as the predicted delta. However, 31 of 38 (81.6%) of the trials had an associated 95% confidence interval that included a delta of 3%, which was the smallest predicted delta sought by investigators in all of the trials [18].

Among all trials, 17 of 38 (44.7%) had an observed delta with a negative value (that is, the treatment was numerically worse than the comparator). Three of these trials showed a statistically significant increase in mortality with the therapy, and all of these trials were stopped early for harm [4,21,22]. The seven trials showing a statistically significant difference favoring the therapy had a smaller delta-gap compared with non-significant trials and those demonstrating harm (delta-gap 0.9% versus 10.5%; *P *< 0.0001). In Figure 1, these seven trials are represented by red triangles above zero on the Y-axis; as can be seen graphically, the deltas associated with these trials fall closer to the blue unity line than the other trials.

For the eight trials that showed a non-statistically significant point estimate for delta that exceeded the smallest predicted delta of all trials (3% [18]), we calculated the sample size that would be required to repeat the study using the observed delta of the index study as the predicted delta for the repeat study. Repeating these trials in this fashion would require increases in sample size from 380% to 1,100% compared with the sample size of the index study (data not shown).

## Discussion

We found that randomized controlled trials of therapies in critical care medicine evaluating proportional mortality as a primary endpoint and published in five high-impact medical journals during the past 10 years utilized predicted values of delta in power calculations that systematically overestimated observed values of delta. We propose that this phenomenon of 'delta inflation' represents a bias in the design of such trials with attendant implications for the design of future trials and the practice of critical care medicine.

Our results accord with the findings of a recent report that found low rates of efficacy in trials in critical care medicine, a finding the authors attributed to the use of mortality as an endpoint [1]. We extend this work by identifying a key feature of such trials, namely that the predicted delta almost uniformly over-estimates the observed delta. This phenomenon of 'delta inflation' is a possible reason that many of these trials fail to demonstrate efficacy. Other investigators have found discrepancies between predicted and observed delta in other fields and with other outcomes, but the overall prevalence of delta inflation in clinical investigation is unknown [23,24]. Our study also complements reports showing that sample size calculations are inadequately or disingenuously reported in randomized controlled trials [8,25,26]. It expands this work by demonstrating that even when there is adequate reporting of statistical methodology, one component of sample size estimation is biased, thus rendering the entire procedure unreliable [9].

The reasons for the discrepancy between predicted and observed delta cannot be determined from our data, but beg speculation. One possibility is that investigators are choosing delta based on sample size rather than choosing sample size based on delta [8,11]. Another possibility is that investigators are overly optimistic about the efficacy and effect size of a therapy and that delta inflation is borne of unrealistic optimism [27]. There may also be a belief that effect sizes below some threshold (say, 10%) are not clinically important, but this is a notion undermined by investigations that sought predicted delta values as low as 3% and by other evidence [18,28]. Moreover, although it has been suggested that delta should be based on an assessment of the MCID, our finding of wide variation in predicted deltas in studies with the same primary outcome demonstrates that this is not happening [29-31]. Publication bias affecting pilot trials may cause those with smaller effect sizes to go unpublished, thereby inflating the apparent benefit of a therapy when and if a literature search is performed [32]; however, the low rate of referenced justification for predicted delta that we and others have documented argues against this [24,33,34]. The insistence on mortality as the gold standard outcome measure in critical care research combined with funding constraints may pressure investigators to search for unrealistic mortality benefits and perhaps to hope that significant improvements in secondary outcome measures will lead to adoption of the therapy [35,36]. Indeed, the very concept of power and the so-called 'double-significance' approach to hypothesis testing and sample size determination has been called into question [37]. Finally, a looming possibility is that the null hypothesis is true and most therapies for critical illness simply are not efficacious. Given the wide confidence intervals around observed delta in the trials in our analysis, this is impossible to disprove with existing data. However, the consistent conduct of trials of therapies that are in reality not efficacious basically would consist of an extreme form of delta inflation. In any case, investigators should take stock in the fact that deltas of 10% or greater are rarely found, and attention needs to be refocused on what is the minimal clinically important difference in trials of therapies to reduce mortality in critical illness [9,31].

Regardless of the causes of delta inflation, its effects are likely deleterious. Firstly, some authors have argued that underpowered trials are unethical and trials designed with delta inflation are essentially underpowered [38]. Secondly, insomuch as delta inflation leads to trials that are 'negative', it may contribute to the premature abandonment of promising therapies because of the commonly held belief that 'absence of evidence is evidence of absence' [39]. This is compounded by the fact that delta inflation can conceal the low statistical power of a trial, thus falsely assuring clinicians that a true difference has been ruled out by a trial with a low type II error rate. Thirdly, the conduct of trials with delta inflation may represent a waste of resources because it undermines their scientific and clinical validity and value to society.

If delta inflation exists, several approaches might minimize its impact. Firstly, not only should predicted delta be reported [40], but also should it be justified by a referenced review of available evidence or a statement about biological plausibility or the MCID, especially when predicted delta exceeds a nominal value such as 3% [18,24]. Results of trials should report confidence intervals for delta rather than *P *values and should emphasize that the results excluded a difference greater than the upper confidence interval rather than stating that the results failed to find a statistically significant difference [11,13,37]. A 'buffer' to account for delta inflation could be built into power calculations as is now done for anticipated rates of drop out and loss to follow up. Moreover, the use of mortality as the only accepted primary outcome for trials of therapies for critical illness should be reconsidered, because few therapies in critical care are ultimately shown to reduce mortality [1,23]. Consideration might be given to the use of composite [41] or weighted composite [42,43] endpoints in which each part of the composite is weighted according to its relative value. For example, a composite endpoint might be comprised of mortality, renal replacement therapy, mechanical ventilation, non-ambulatory status, or receiving nutritional support at some pre-determined time point (e.g., 28 or 60 days). More research related to long-term outcomes in critical illness and their relative values will be needed to inform the choice of components of composite endpoints [44].

There are several limitations of our study. As we limited our search to five high-impact journals, it is possible that we have overestimated the prevalence of delta inflation because of omission of trials that more accurately predicted delta in other journals. This is unlikely because high-impact journals are more likely to publish 'positive' trials and those with larger sample sizes and larger effects, and thus our analysis may have underestimated the prevalence and impact of delta inflation. For the sake of homogeneity, we limited our analysis to critical care trials that utilized mortality as a primary endpoint, and therefore our findings may not be generalizable to trials in other specialties and those using other primary outcomes. Nonetheless, the same pressures faced by critical care investigators may be experienced by investigators in other fields pursuing other outcomes who may likewise be susceptible to delta inflation. Determination of the prevalence of delta inflation in other arenas will require specific study.

## Conclusions

Delta inflation, a systematic overestimation in predictions of treatment effect size during trial design, is common in randomized controlled trials of mortality in critical care medicine. Reliable methods for predicting delta during study design and better reporting of the basis for these predictions are needed to minimize the risk of trial failure from type II statistical errors and resulting waste of research resources. Consideration should be given to designing such trials with other clinically meaningful primary endpoints. Critical care practitioners and investigators must be aware that because of delta inflation, negative results in randomized controlled trials do not rule out efficacy of the therapies evaluated.

## Key messages

• Most therapies for adult critical illness fail to demonstrate efficacy in randomized controlled trials.

• In the design of randomized controlled trials, investigators must determine a realistic estimate of the effect size (delta) of the therapy on an outcome of interest such as mortality.

• In randomized controlled trials in critical care, predicted delta almost always exceeds the delta observed in the trial data.

• This 'delta inflation' is a potential reason that most such trials fail to demonstrate efficacy.

• Critical care practitioners and investigators must bear in mind that 'absence of evidence is not evidence of absence'.

## Abbreviations

delta: effect size; MCID: minimal clinically important difference.

## Competing interests

The authors declare that they have no competing interests.

## Authors' contributions

SKA conceived the idea for the article, performed the data abstraction and analysis and wrote the manuscript. JMOB assisted with conception of the article and with writing and editing of the manuscript. DRR assisted with data collection and analysis, and analysis plan.

## Supplementary Material

Additional file 1 **Table S2**. Selected characteristics of studies included in the analysis. Click here for file
