# Supplementary material for: Delta inflation: a bias in the design of randomized controlled trials in critical care medicine
Source: Crit Care. 2010 Apr 29;14(2):R77. doi: 10.1186/cc8990 (PMC2887200; doi:10.1186/cc8990)
Supplement: Additional file 1 — Table S2. Selected characteristics of studies included in the analysis. [file cc8990-S1.PDF]

Study Title	Author	Year	Journal	Stopped early?	δ justified?	Predicted δ	Planned Power	Our Power calculation	Observed δ	Lower CI for δ	Upper CI for δ	δ-gap
Diaspirin Cross-Linked Hemoglobin (DCLHb) in the Treatment of Severe Traumatic Hemorrhagic Shock: A Randomized Controlled Efficacy Trial	Sloan[1]	1999	JAMA	Harm	No	0.1	85%	85.0%	-0.220	-0.393	-0.047	0.32
E5 murine monoclonal antiendotoxin antibody in gram-negative sepsis: a randomized controlled trial. E5 Study Investigators.	Angus[2]	2000	JAMA	No	No	0.07	90%	90.3%	0.014	-0.041	0.069	0.056
Ketoconazole for Early Treatment of Acute Lung Injury and Acute Respiratory Distress Syndrome	ARDSnet[3]	2000	JAMA	DSMB	No	0.1	80%	Insufficient data	-0.011	-0.133	0.111	0.111
Invasive and noninvasive strategies for management of suspected ventilator-associated pneumonia. A randomized trial.	Fagon[4]	2000	Annals	No	No	0.1	80%	59.3%	0.096	0.018	0.174	0.004
Effects of different doses in continuous veno-venous haemofiltration on outcomes of acute renal failure: a prospective randomised trial	Ronco[5]	2000	Lancet	No	No	0.2	Not stated	Insufficient data	0.170	0.071	0.269	0.03
Ventilation with lower tidal volumes as compared with traditional tidal voumes for acute lung injury and the acute respiratory distress syndrome	Brower[6]	2000	NEJM	Yes	No	0.1[1]	Not stated	Insufficient data	0.088	0.024	0.152	0.012
Effect of prone positioning on the survival of patients with acute respiratory failure.	Gattinoni[7]	2001	NEJM	Declining enrollment	No	0.05	80%	Insufficient data	0.040	-0.055	0.135	0.01
High-dose antithrombin III in severe sepsis: a randomized controlled trial.	Warren[8]	2001	JAMA	No	No	0.0675	85%	90.1%	-0.002	-0.042	0.038	0.0695
Intensive insulin therapy in critically ill patients.	Van Den Berghe[9]	2001	NEJM	Benefit	No	0.05	Not stated	Insufficient data	0.034	0.010	0.058	0.016

[1] provided by Roy G. Brower, MD in a personal communication

Early goal-directed therapy in the treatment of severe sepsis and septic shock.	Rivers[10]	2001	nejm	No	No	0.15	80%	Insufficient data	0.160	0.044	0.276	-0.01
Efficacy and safety of recombinant human protein C for severe sepsis	Bernard[11]	2001	NEJM	Yes	No	0.054[2]	Not stated	Insufficient data	0.061	0.018	0.104	-0.007
Effect of treatment with low doses of hydrocortisone and fludrocortisone on mortality in patients with septic shock	Annane[12]	2002	JAMA	No	Yes	0.2	90%	99.8%[3]	0.060	-0.052	0.172	0.14
Daily hemodialysis and the outcome of acute renal failure	Schiffl[13]	2002	NEJM	No	No	0.2	0.8	70.5%	0.180	0.026	0.334	0.02
Early use of the pulmonary artery catheter and outcomes in patients with shock and acute respiratory distress syndrome: a randomized controlled trial.	Richard[14]	2003	JAMA	Declining enrollment	No	0.1	78%[4]	Insufficient data	0.016	-0.058	0.090	0.084
Higher versus Lower Positive End-Expiratory Pressures in Patients with the Acute Respiratory Distress Syndrome	ARDSnet[15]	2004	NEJM	Futility	No	0.1	89%	88.8%	-0.028	-0.101	0.045	0.128
Effects of systematic prone positioning in hypoxemic acute respiratory failure: a randomized controlled trial	Guerin[16]	2004	JAMA	No	No	0.1	80%	80.0%	-0.009	-0.074	0.056	0.109
A Comparison of Albumin and Saline for Fluid Resuscitation in the Intensive Care Unit	SAFE investigators[17]	2004	NEJM	No	No	0.03	90%	95.3%	0.002	-0.017	0.021	0.028
Noninvasive positive-pressure ventilation for respiratory failure after extubation	Esteban[18]	2004	NEJM	Harm	No	0.13	80%	Insufficient data	-0.110	-0.213	-0.007	0.24
Drotrecogin alfa (activated) for adults with severe sepsis and a low risk of death	Abraham[19]	2005	NEJM	Futility	No	0.04	90%[5]	100.0%	-0.015	-0.044	0.014	0.055

[2] provided by Howard Levy, MD, PhD in a personal communication
[3] based on one-sided α as reported in the manuscript
[4] a priori power was initially 90%, revised mid-trial to 80%; final power 78% at the time of early termination of the trial due to declining enrollment
[5] the authors reported that the trial was designed to have "*at least* 90% power to detect a statistically significant difference"

Assessment of the clinical effectiveness of pulmonary artery catheters in management of patients in intensive care (PAC-Man): a randomised controlled trial	Harvey[20]	2005	Lancet	No	No	0.1[6]	90%	94.4%	-0.020	-0.078	0.038	0.12
A Randomized Trial of Diagnostic Techniques for Ventilator-Associated Pneumonia	Canadian Critical Care Trials Group[21]	2006	NEJM	No	Yes	0.1	80%	79.1%	-0.005	-0.061	0.051	0.105
Efficacy and Safety of Corticosteroids for Persistent Acute Respiratory Distress Syndrome	NHLBI ARDSnet[22]	2006	NEJM	No	No	0.2[7]	85%	79.5%	-0.006	-0.138	0.126	0.206
Comparison of Two Fluid-Management Strategies in Acute Lung Injury	NHLBI ARDSnet[23]	2006	NEJM	No	No	0.1	90%	94.3%	0.029	-0.026	0.084	0.071
Pulmonary-Artery versus Central Venous Catheter to Guide Treatment of Acute Lung Injury	NHLBI ARDSnet[24]	2006	NEJM	No	No	0.1	90%	Insufficient data	-0.011	-0.066	0.044	0.111
Intensive insulin therapy in the medical ICU	Van Den Berghe[25]	2006	NEJM	No	Yes	0.07[8]	80%	Insufficient data	0.026	-0.023	0.075	0.044
Continuous venovenous haemodiafiltration versus intermittent haemodialysis for acute renal failure in patients with multiple-organ dysfunction syndrome: a multicentre randomised trial.	Vinsonneau[26]	2006	Lancet	No	No	0.15	90%	81.1%	0.029	-0.072	0.130	0.121
Norepinephrine plus dobutamine versus epinephrine alone for management of septic shock: a randomised trial.	Annane[27]	2007	Lancet	No	No	0.2	95%	95.1%	-0.060	-0.164	0.044	0.26
**Intensive insulin** therapy and pentastarch resuscitation in severe sepsis	Brunkhorst[28]	2008	NEJM	Harm	No	0.1	80%	70.0%	0.013	-0.061	0.087	0.087

[6] the initial pre-specified δ was stated to be 5%, but was revised mid-trial to 10% due to low enrollment
[7] the initial pre-specified δ was 15%, but was revised mid-trial to 20% due to low enrollment
[8] this δ is pre-specified for the subgroup of patients who remain in the ICU for >3 days, but was used to calculate the sample size for the overall intention-to-treat population

Intensive insulin therapy and **pentastarch** resuscitation in severe sepsis	Brunkhorst[28a]	2008	NEJM	Harm	No	0.1	80%	70.0%	-0.026	-0.100	0.048	0.126
Effect of Evidence-Based Feeding Guidelines on Mortality of Critically Ill Adults: A Cluster Randomized Controlled Trial	Doig[29]	2008	JAMA	No	Yes	0.08	80%	92.3%[9]	-0.015	-0.068	0.038	0.095
Ventilation strategy using low tidal volumes, recruitment maneuvers, and high positive end-expiratory pressure for acute lung injury and acute respiratory distress syndrome: a randomized controlled trial.	Meade[30]	2008	JAMA	No	No	0.09	80%	80.2%	0.040	-0.021	0.101	0.05
Positive end-expiratory pressure setting in adults with acute lung injury and acute respiratory distress syndrome: a randomized controlled trial	Mercat[31]	2008	JAMA	No	No	0.1	80%	82.5%	0.034	-0.031	0.099	0.066
Vasopressin versus norepinephrine infusion in patients with septic shock	Russell[32]	2008	NEJM	No	No	0.1	80%	78.0%	0.039	-0.029	0.107	0.061
Hydrocortisone therapy for patients with septic shock	Sprung[33]	2008	NEJM	No	No	0.1	80%	79.3%	-0.028	-0.110	0.054	0.128
Intensity of Renal Support in Critically Ill Patients with Acute Kidney Injury	VA-NIH ARFTN[34]	2008	NEJM	No	No	0.1	90%	91.9%	-0.021	-0.079	0.037	0.121
Decontamination of the **Digestive Tract** and Oropharynx in ICU Patients	DeSmet[35]	2009	NEJM	No	No	0.04	80%	87.6%	0.006	-0.021	0.033	0.034
Decontamination of the Digestive Tract and **Oropharynx** in ICU Patients	DeSmet[35a]	2009	NEJM	No	No	0.04	80%	87.6%	0.009	-0.019	0.037	0.031
Intensive versus Conventional Glucose Control in Critically Ill Patients	NICE SUGAR[36]	2009	NEJM	Harm	Yes	0.038	90%	90.6%	-0.026	-0.048	-0.004	0.064

[9] our calculations using conventional methods do not account for intracluster correlations

Table 2. Selected characteristics of studies included in the analysis.

Reference List

1. Sloan EP, Koenigsberg M, Gens D et al. Diaspirin Cross-Linked Hemoglobin (DCLHb) in the Treatment of Severe Traumatic Hemorrhagic Shock: A Randomized Controlled Efficacy Trial. JAMA 1999;282(19):1857-1864.

2. Angus DC, Birmingham MC, Balk RA et al. E5 murine monoclonal antiendotoxin antibody in gram-negative sepsis: a randomized controlled trial. E5 Study Investigators. JAMA 2000;283(13):1723-1730.

3. The ARDS Network Authors for the ARDS Network. Ketoconazole for Early Treatment of Acute Lung Injury and Acute Respiratory Distress Syndrome: A Randomized Controlled Trial. JAMA 2000;283(15):1995-2002.

4. Fagon JY, Chastre J, Wolff M et al. Invasive and noninvasive strategies for management of suspected ventilator-associated pneumonia. A randomized trial. Ann Intern Med 2000;132(8):621-630.

5. Ronco C, Bellomo R, Homel P et al. Effects of different doses in continuous veno-venous haemofiltration on outcomes of acute renal failure: a prospective randomised trial. Lancet 2000;356(9223):26-30.

6. Ventilation with lower tidal volumes as compared with traditional tidal volumes for acute lung injury and the acute respiratory distress syndrome. The Acute Respiratory Distress Syndrome Network. N Engl J Med 2000;342(18):1301-1308.

7. Gattinoni L, Tognoni G, Pesenti A et al. Effect of Prone Positioning on the Survival of Patients with Acute Respiratory Failure. N Engl J Med 2001;345(8):568-573.

8. Warren BL, Eid A, Singer P et al. Caring for the critically ill patient. High-dose antithrombin III in severe sepsis: a randomized controlled trial. JAMA 2001;286(15):1869-1878.

9. Van den Berghe G, Wouters P, Weekers F et al. Intensive Insulin Therapy in Critically Ill Patients. N Engl J Med 2001;345(19):1359-1367.

10. Rivers E, Nguyen B, Havstad S et al. Early Goal-Directed Therapy in the Treatment of Severe Sepsis and Septic Shock. N Engl J Med 2001;345(19):1368-1377.

11. Bernard GR, Vincent JL, Laterre PF et al. Efficacy and Safety of Recombinant Human Activated Protein C for Severe Sepsis. N Engl J Med 2001;344(10):699-709.

12. Annane D, Sebille V, Charpentier C et al. Effect of Treatment With Low Doses of Hydrocortisone and Fludrocortisone on Mortality in Patients With Septic Shock. JAMA 2002;288(7):862-871.

13. Schiffl H, Lang SM, Fischer R. Daily Hemodialysis and the Outcome of Acute Renal Failure. N Engl J Med 2002;346(5):305-310.

14. Richard C, Warszawski J, Anguel N et al. Early Use of the Pulmonary Artery Catheter and Outcomes in Patients With Shock and Acute Respiratory Distress Syndrome: A Randomized Controlled Trial. JAMA 2003;290(20):2713-2720.

15. The National Heart LaBIACTN. Higher versus Lower Positive End-Expiratory Pressures in Patients with the Acute Respiratory Distress Syndrome. N Engl J Med 2004;351(4):327-336.

16. Guerin C, Gaillard S, Lemasson S et al. Effects of Systematic Prone Positioning in Hypoxemic Acute Respiratory Failure: A Randomized Controlled Trial. JAMA 2004;292(19):2379-2387.

17. The SS, I. A Comparison of Albumin and Saline for Fluid Resuscitation in the Intensive Care Unit. N Engl J Med 2004;350(22):2247-2256.

18. Esteban A, Anzueto A, Frutos F et al. Characteristics and Outcomes in Adult Patients Receiving Mechanical Ventilation: A 28-Day International Study. JAMA 2002;287(3):345-355.

19. Abraham E, Laterre PF, Garg R et al. Drotrecogin Alfa (Activated) for Adults with Severe Sepsis and a Low Risk of Death. N Engl J Med 2005;353(13):1332-1341.

20. Harvey S, Harrison DA, Singer M et al. Assessment of the clinical effectiveness of pulmonary artery catheters in management of patients in intensive care (PAC-Man): a randomised controlled trial. Lancet 2005;366(9484):472-477.

21. the Canadian Critical Care Trials Group. A Randomized Trial of Diagnostic Techniques for Ventilator-Associated Pneumonia. N Engl J Med 2006;355(25):2619-2630.

22. The National Heart LaBIARDSACTN. Efficacy and Safety of Corticosteroids for Persistent Acute Respiratory Distress Syndrome. N Engl J Med 2006;354(16):1671-1684.

23. Wiedemann HP, Wheeler AP, Bernard GR et al. Comparison of two fluid-management strategies in acute lung injury. N Engl J Med 2006;354(24):2564-2575.

24. The National Heart LaBIARDSACTN. Pulmonary-Artery versus Central Venous Catheter to Guide Treatment of Acute Lung Injury. N Engl J Med 2006;354(21):2213-2224.

25. Van den Berghe G, Wilmer A, Hermans G et al. Intensive Insulin Therapy in the Medical ICU. N Engl J Med 2006;354(5):449-461.

26. Vinsonneau C, Camus C, Combes A et al. Continuous venovenous haemodiafiltration versus intermittent haemodialysis for acute renal failure in patients with multiple-organ dysfunction syndrome: a multicentre randomised trial. Lancet 2006;368(9533):379-385.

27. Annane D, Vignon P, Renault A et al. Norepinephrine plus dobutamine versus epinephrine alone for management of septic shock: a randomised trial. Lancet 2007;370(9588):676-684.

28. Brunkhorst FM, Engel C, Bloos F et al. Intensive Insulin Therapy and Pentastarch Resuscitation in Severe Sepsis. N Engl J Med 2008;358(2):125-139.

29. Doig GS, Simpson F, Finfer S et al. Effect of Evidence-Based Feeding Guidelines on Mortality of Critically Ill Adults: A Cluster Randomized Controlled Trial. JAMA 2008;300(23):2731-2741.

30. Meade MO, Cook DJ, Guyatt GH et al. Ventilation Strategy Using Low Tidal Volumes, Recruitment Maneuvers, and High Positive End-Expiratory Pressure for Acute Lung Injury and Acute Respiratory Distress Syndrome: A Randomized Controlled Trial. JAMA 2008;299(6):637-645.

31. Mercat A, Richard JC, Vielle B et al. Positive End-Expiratory Pressure Setting in Adults With Acute Lung Injury and Acute Respiratory Distress Syndrome: A Randomized Controlled Trial. JAMA 2008;299(6):646-655.

32. Russell JA, Walley KR, Singer J et al. Vasopressin versus Norepinephrine Infusion in Patients with Septic Shock. N Engl J Med 2008;358(9):877-887.

33. Sprung CL, Annane D, Keh D et al. Hydrocortisone Therapy for Patients with Septic Shock. N Engl J Med 2008;358(2):111-124.

34. The VA/NIH Acute Renal Failure Trial Network. Intensity of Renal Support in Critically Ill Patients with Acute Kidney Injury. N Engl J Med 2008;359(1):7-20.

35. de Smet AMGA, Kluytmans JAJW, Cooper BS et al. Decontamination of the Digestive Tract and Oropharynx in ICU Patients. N Engl J Med 2009;360(1):20-31.

36. The NICE-SUGAR Study Investigators. Intensive versus Conventional Glucose Control in Critically Ill Patients. N Engl J Med 2009;360(13):1283-1297.
